# (1*E*,1′*E*)-4,4′-[1,1′-(Hydrazine-1,2-diyl­idene)bis­(ethan-1-yl-1-yl­idene)]diphenol dihydrate

**DOI:** 10.1107/S1600536811029679

**Published:** 2011-08-02

**Authors:** Suchada Chantrapromma, Patcharaporn Jansrisewangwong, Kullapa Chanawanno, Hoong-Kun Fun

**Affiliations:** a Crystal Materials Research Unit, Department of Chemistry, Faculty of Science, Prince of Songkla University, Hat-Yai, Songkhla 90112, Thailand; b Department of Chemistry and Center of Excellence for Innovation in Chemistry, Faculty of Science, Prince of Songkla University, Hat-Yai, Songkhla 90112, Thailand; c X-ray Crystallography Unit, School of Physics, Universiti Sains Malaysia, 11800 USM, Penang, Malaysia

## Abstract

The asymmetric unit of the title compound, C_16_H_16_N_2_O_2_·2H_2_O, contains one half-mol­ecule of diphenol and one water mol­ecule. The complete diphenol mol­ecule is generated by a crystallographic inversion centre. In the mol­ecule, the central C_meth­yl_—C=N—N=C—C_meth­yl_ plane makes a dihedral angle of 8.88 (6)° with its adjacent benzene ring. In the crystal, the components are linked by O—H⋯N and O—H⋯O hydrogen bonds into a three-dimensional network. The crystal structure is further stabilized by a weak C—H⋯π inter­action.

## Related literature

For bond-length data, see: Allen *et al.* (1987 ▶). For related structures, see: Chantrapromma *et al.* (2010 ▶); Fun *et al.* (2010 ▶); Jansrisewangwong *et al.* (2010 ▶). For background to and the biological activity of hydro­zones, see: Bendre *et al.* (1998 ▶); El-Tabl *et al.* (2008 ▶); Kitaev *et al.* (1970 ▶); Qin *et al.* (2009 ▶); Ramamohan *et al.* (1995 ▶); Rollas & Küçükgüzel (2007 ▶). For the stability of the temperature controller used in the data collection, see: Cosier & Glazer (1986 ▶).
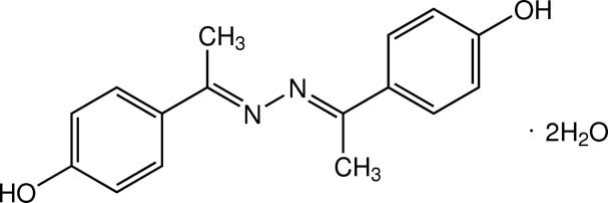


## Experimental

### 

#### Crystal data

C_16_H_16_N_2_O_2_·2H_2_O
*M* *_r_* = 304.34
Monoclinic, 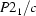

*a* = 7.8522 (1) Å
*b* = 5.5151 (1) Å
*c* = 17.8918 (3) Å
β = 108.536 (1)°
*V* = 734.62 (2) Å^3^
*Z* = 2
Mo *K*α radiation
μ = 0.10 mm^−1^
*T* = 100 K
0.35 × 0.26 × 0.22 mm

#### Data collection

Bruker APEXII CCD area-detector diffractometer
Absorption correction: multi-scan (*SADABS*; Bruker, 2005 ▶) *T*_min_ = 0.966, *T*_max_ = 0.979
8010 measured reflections
2129 independent reflections
1903 reflections with *I* > 2σ(*I*)
*R*_int_ = 0.021

#### Refinement

*R*[*F* ^2^ > 2σ(*F* ^2^)] = 0.046
*wR*(*F* ^2^) = 0.125
*S* = 1.06
2129 reflections
101 parameters
H-atom parameters constrained
Δρ_max_ = 0.39 e Å^−3^
Δρ_min_ = −0.34 e Å^−3^

### 

Data collection: *APEX2* (Bruker, 2005 ▶); cell refinement: *SAINT* (Bruker, 2005 ▶); data reduction: *SAINT*; program(s) used to solve structure: *SHELXTL* (Sheldrick, 2008 ▶); program(s) used to refine structure: *SHELXTL*; molecular graphics: *SHELXTL*; software used to prepare material for publication: *SHELXTL* and *PLATON* (Spek, 2009 ▶).

## Supplementary Material

Crystal structure: contains datablock(s) global, I. DOI: 10.1107/S1600536811029679/is2750sup1.cif

Structure factors: contains datablock(s) I. DOI: 10.1107/S1600536811029679/is2750Isup2.hkl

Supplementary material file. DOI: 10.1107/S1600536811029679/is2750Isup3.cml

Additional supplementary materials:  crystallographic information; 3D view; checkCIF report

## Figures and Tables

**Table 1:** Hydrogen-bond geometry (Å, °) *Cg*1 is the centroid of the C1–C6 ring.

*D*—H⋯*A*	*D*—H	H⋯*A*	*D*⋯*A*	*D*—H⋯*A*
O1—H1*O*1⋯O1*W*^i^	0.83	1.86	2.6747 (12)	171
O1*W*—H1*W*⋯O1^ii^	0.86	2.07	2.8429 (12)	149
O1*W*—H2*W*⋯N1^iii^	0.86	2.17	3.0132 (14)	166
C5—H5*A*⋯*Cg*1^iv^	0.93	2.80	3.5046 (12)	134

Symmetry codes: (i) 
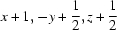
; (ii) 
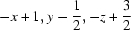
; (iii) 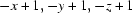; (iv) 
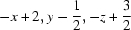
.

**Fig. 1.**
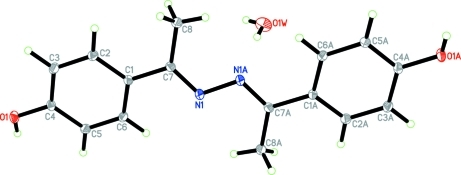
The molecular structure of the title compound, showing 50% probability displacement ellipsoids and the atom-numbering scheme. Atoms with suffix A were generated by symmetry code 1 - x, 1 - y, 1 - z.

**Fig. 2.**
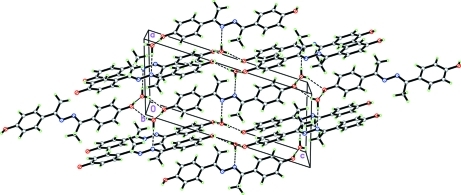
The crystal packing of the title compound viewed approximately along the b axis, showing three dimensional network.

## supplementary crystallographic information

### Comment

Hydrazones have been reported to possess fluorescence properties (Qin *et al.*, 2009) and various biological activities such as to be used as insecticides, antitumor agents and antioxidants (Kitaev *et al.*, 1970), as well as antimicrobial (Ramamohan *et al.*, 1995) and antiviral properties (El-Tabl *et al.*, 2008; Rollas & Küçükgüzel, 2007) and tyrosinase inhibitory activity (Bendre *et al.*, 1998). With our on-going research on structural studies and properties of hydrazones (Chantrapromma *et al.*, 2010; Fun *et al.*, 2010; Jansrisewangwong *et al.*, 2010), the title compound (I) was synthesized. Our results show that (I) was inactive for tyrosinase inhibitory activity. Herein we report the synthesis and crystal structure of the title compound (I).

The asymmetric unit of (I) (Fig. 1), C_16_H_16_N_2_O_2_.2H_2_O, contains one half-molecule of diphenol and the complete molecule is generated by a crystallographic inversion centre 1 - *x*, 1 - *y*, 1 - *z*. The molecule of (I) exists in an *E*,*E* configuration with respect to the two C═N double bonds [1.2985 (13) Å] and the torsion angle N1A–N1–C7–C1 = 177.76 (10)°. The diethylidenehydrazine moiety (C7/C8/N1/N1A/C7A/C8A) is planar with an *r.m.s* deviation of 0.0084 (1) Å. This C/C/N/N/C/C plane makes a dihedral angle of 8.88 (6)° with its both adjacent benzene rings. Each hydroxy group is co-planarly attached with the benzene ring with the *r.m.s*. of 0.0056 (1) Å for the seven non H atoms. The bond distances are of normal values (Allen *et al.*, 1987) and are comparable with related structures (Chantrapromma *et al.*, 2010; Fun *et al.*, 2010; Jansrisewangwong *et al.*, 2010).

In the crystal structure (Fig. 2), the molecules are linked into three dimensional network by O—H···N and O—H···O hydrogen bonds (Table 1). C—H···π interaction was also also observed (Table 1).

### Experimental

The title compound was synthesized by mixing a solution (1:2 molar ratio) of hydrazine hydrate (0.10 ml, 2 mmol) and 4-hydroxyacetophenone (0.54 g, 4 mmol) in ethanol (20 ml). The resulting solution was refluxed for 6 h, yielding the yellow solid. The resultant solid was filtered off and washed with methanol. Yellow block-shaped single crystals of the title compound suitable for *x*-ray structure determination were recrystalized from acetone by slow evaporation of the solvent at room temperature over several days, m.p. 377–379 K.

### Refinement

The water hydrogen atoms were restrained to the ideal positions. The remaining H atoms were positioned geometrically and allowed to ride on their parent atoms, with *d*(O—H) = 0.86 Å, and *d*(C—H) = 0.93 Å for aromatic and 0.96 Å for CH_3_ atoms. The *U*_iso_ values were constrained to be 1.5*U*_eq_ of the carrier atom for methyl H atoms and 1.2*U*_eq_ for the remaining H atoms. A rotating group model was used for the methyl groups. The highest residual electron density peak is located at 0.40 Å from H1W and the deepest hole is located at 0.35 Å from H1W.

### Crystal data

**Table:**

C_16_H_16_N_2_O_2_·2H_2_O	*F*(000) = 324
*M**_r_* = 304.34	*D*_x_ = 1.376 Mg m^−^^3^
Monoclinic, *P*2_1_/*c*	Melting point = 377–379 K
Hall symbol: -P 2ybc	Mo *K*α radiation, λ = 0.71073 Å
*a* = 7.8522 (1) Å	Cell parameters from 2129 reflections
*b* = 5.5151 (1) Å	θ = 2.4–30.0°
*c* = 17.8918 (3) Å	µ = 0.10 mm^−^^1^
β = 108.536 (1)°	*T* = 100 K
*V* = 734.62 (2) Å^3^	Block, yellow
*Z* = 2	0.35 × 0.26 × 0.22 mm

### Data collection

**Table:**

Bruker APEXII CCD area-detector diffractometer	2129 independent reflections
Radiation source: sealed tube	1903 reflections with *I* > 2σ(*I*)
graphite	*R*_int_ = 0.021
φ and ω scans	θ_max_ = 30.0°, θ_min_ = 2.4°
Absorption correction: multi-scan (*SADABS*; Bruker, 2005)	*h* = −10→11
*T*_min_ = 0.966, *T*_max_ = 0.979	*k* = −7→7
8010 measured reflections	*l* = −24→24

### Refinement

**Table:**

Refinement on *F*^2^	Primary atom site location: structure-invariant direct methods
Least-squares matrix: full	Secondary atom site location: difference Fourier map
*R*[*F*^2^ > 2σ(*F*^2^)] = 0.046	Hydrogen site location: inferred from neighbouring sites
*wR*(*F*^2^) = 0.125	H-atom parameters constrained
*S* = 1.06	*w* = 1/[σ^2^(*F*_o_^2^) + (0.061*P*)^2^ + 0.4114*P*] where *P* = (*F*_o_^2^ + 2*F*_c_^2^)/3
2129 reflections	(Δ/σ)_max_ = 0.001
101 parameters	Δρ_max_ = 0.39 e Å^−^^3^
0 restraints	Δρ_min_ = −0.34 e Å^−^^3^

### Special details

**Table:**

Experimental. The crystal was placed in the cold stream of an Oxford Cryosystems Cobra open-flow nitrogen cryostat (Cosier & Glazer, 1986) operating at 120.0 (1) K.
Geometry. All e.s.d.'s (except the e.s.d. in the dihedral angle between two l.s. planes) are estimated using the full covariance matrix. The cell e.s.d.'s are taken into account individually in the estimation of e.s.d.'s in distances, angles and torsion angles; correlations between e.s.d.'s in cell parameters are only used when they are defined by crystal symmetry. An approximate (isotropic) treatment of cell e.s.d.'s is used for estimating e.s.d.'s involving l.s. planes.
Refinement. Refinement of *F*^2^ against ALL reflections. The weighted *R*-factor *wR* and goodness of fit *S* are based on *F*^2^, conventional *R*-factors *R* are based on *F*, with *F* set to zero for negative *F*^2^. The threshold expression of *F*^2^ > σ(*F*^2^) is used only for calculating *R*-factors(gt) *etc*. and is not relevant to the choice of reflections for refinement. *R*-factors based on *F*^2^ are statistically about twice as large as those based on *F*, and *R*- factors based on ALL data will be even larger.

### Fractional atomic coordinates and isotropic or equivalent isotropic displacement parameters (Å^2^)

**Table:**

	*x*	*y*	*z*	*U*_iso_*/*U*_eq_	
O1	0.97567 (10)	0.63439 (15)	0.90238 (4)	0.01443 (19)	
H1O1	1.0145	0.4971	0.9166	0.022*	
N1	0.55899 (11)	0.49760 (17)	0.53887 (5)	0.0144 (2)	
C1	0.64270 (13)	0.65293 (19)	0.66844 (6)	0.0106 (2)	
C2	0.63628 (14)	0.83658 (19)	0.72163 (6)	0.0131 (2)	
H2A	0.5566	0.9650	0.7045	0.016*	
C3	0.74735 (14)	0.82980 (19)	0.79977 (6)	0.0137 (2)	
H3A	0.7421	0.9534	0.8343	0.016*	
C4	0.86615 (13)	0.63756 (19)	0.82596 (6)	0.0111 (2)	
C5	0.87307 (13)	0.45094 (19)	0.77420 (6)	0.0126 (2)	
H5A	0.9515	0.3215	0.7918	0.015*	
C6	0.76265 (13)	0.45958 (19)	0.69653 (6)	0.0123 (2)	
H6A	0.7680	0.3350	0.6623	0.015*	
C7	0.52532 (13)	0.65863 (19)	0.58524 (6)	0.0113 (2)	
C8	0.37991 (14)	0.8466 (2)	0.56043 (6)	0.0160 (2)	
H8A	0.4326	1.0054	0.5696	0.024*	
H8B	0.2989	0.8269	0.5904	0.024*	
H8C	0.3152	0.8278	0.5054	0.024*	
O1W	0.07930 (12)	0.31062 (17)	0.45773 (6)	0.0252 (2)	
H1W	0.0793	0.3113	0.5058	0.038*	
H2W	0.1722	0.3860	0.4541	0.038*	

### Atomic displacement parameters (Å^2^)

**Table:**

	*U*^11^	*U*^22^	*U*^33^	*U*^12^	*U*^13^	*U*^23^
O1	0.0167 (4)	0.0153 (4)	0.0088 (3)	0.0003 (3)	0.0007 (3)	0.0002 (3)
N1	0.0136 (4)	0.0180 (5)	0.0093 (4)	0.0038 (3)	0.0002 (3)	−0.0019 (3)
C1	0.0106 (4)	0.0118 (5)	0.0093 (4)	−0.0001 (3)	0.0029 (3)	−0.0004 (3)
C2	0.0145 (4)	0.0122 (5)	0.0121 (4)	0.0025 (4)	0.0034 (4)	−0.0005 (3)
C3	0.0163 (5)	0.0125 (5)	0.0120 (4)	0.0007 (4)	0.0041 (4)	−0.0025 (3)
C4	0.0114 (4)	0.0131 (5)	0.0087 (4)	−0.0023 (3)	0.0030 (3)	−0.0001 (3)
C5	0.0132 (4)	0.0125 (5)	0.0116 (4)	0.0026 (3)	0.0033 (3)	0.0004 (3)
C6	0.0141 (4)	0.0121 (5)	0.0106 (4)	0.0013 (3)	0.0039 (3)	−0.0017 (3)
C7	0.0103 (4)	0.0131 (5)	0.0102 (4)	0.0005 (3)	0.0028 (3)	0.0006 (3)
C8	0.0164 (5)	0.0166 (5)	0.0128 (5)	0.0059 (4)	0.0016 (4)	−0.0010 (4)
O1W	0.0194 (4)	0.0227 (5)	0.0337 (5)	−0.0016 (3)	0.0089 (4)	0.0060 (4)

### Geometric parameters (Å, °)

**Table:**

O1—C4	1.3644 (11)	C4—C5	1.3971 (14)
O1—H1O1	0.8256	C5—C6	1.3854 (13)
N1—C7	1.2985 (13)	C5—H5A	0.9300
N1—N1^i^	1.4050 (16)	C6—H6A	0.9300
C1—C2	1.4016 (14)	C7—C8	1.5010 (14)
C1—C6	1.4056 (14)	C8—H8A	0.9600
C1—C7	1.4814 (13)	C8—H8B	0.9600
C2—C3	1.3934 (13)	C8—H8C	0.9600
C2—H2A	0.9300	O1W—H1W	0.8598
C3—C4	1.3913 (14)	O1W—H2W	0.8601
C3—H3A	0.9300		
			
C4—O1—H1O1	111.9	C6—C5—H5A	120.1
C7—N1—N1^i^	114.55 (10)	C4—C5—H5A	120.1
C2—C1—C6	118.04 (9)	C5—C6—C1	121.28 (9)
C2—C1—C7	121.45 (9)	C5—C6—H6A	119.4
C6—C1—C7	120.50 (9)	C1—C6—H6A	119.4
C3—C2—C1	121.04 (9)	N1—C7—C1	116.07 (9)
C3—C2—H2A	119.5	N1—C7—C8	125.01 (9)
C1—C2—H2A	119.5	C1—C7—C8	118.92 (9)
C4—C3—C2	119.82 (9)	C7—C8—H8A	109.5
C4—C3—H3A	120.1	C7—C8—H8B	109.5
C2—C3—H3A	120.1	H8A—C8—H8B	109.5
O1—C4—C3	119.26 (9)	C7—C8—H8C	109.5
O1—C4—C5	120.65 (9)	H8A—C8—H8C	109.5
C3—C4—C5	120.09 (9)	H8B—C8—H8C	109.5
C6—C5—C4	119.72 (9)	H1W—O1W—H2W	110.1
			
C6—C1—C2—C3	0.95 (16)	C2—C1—C6—C5	−0.70 (15)
C7—C1—C2—C3	−179.93 (9)	C7—C1—C6—C5	−179.83 (9)
C1—C2—C3—C4	−0.37 (16)	N1^i^—N1—C7—C1	177.76 (10)
C2—C3—C4—O1	179.26 (9)	N1^i^—N1—C7—C8	−2.78 (17)
C2—C3—C4—C5	−0.48 (16)	C2—C1—C7—N1	171.18 (10)
O1—C4—C5—C6	−179.01 (9)	C6—C1—C7—N1	−9.72 (14)
C3—C4—C5—C6	0.72 (15)	C2—C1—C7—C8	−8.31 (15)
C4—C5—C6—C1	−0.12 (16)	C6—C1—C7—C8	170.78 (10)

Symmetry codes: (i) −*x*+1, −*y*+1, −*z*+1.

### Hydrogen-bond geometry (Å, °)

**Table:**

*Cg*1 is the centroid of the C1–C6 ring.

**Table:**

*D*—H···*A*	*D*—H	H···*A*	*D*···*A*	*D*—H···*A*
O1—H1O1···O1W^ii^	0.83	1.86	2.6747 (12)	171
O1W—H1W···O1^iii^	0.86	2.07	2.8429 (12)	149
O1W—H2W···N1^i^	0.86	2.17	3.0132 (14)	166
C5—H5A···Cg1^iv^	0.93	2.80	3.5046 (12)	134

Symmetry codes: (ii) *x*+1, −*y*+1/2, *z*+1/2; (iii) −*x*+1, *y*−1/2, −*z*+3/2; (i) −*x*+1, −*y*+1, −*z*+1; (iv) −*x*+2, *y*−1/2, −*z*+3/2.
